# Chiari malformation type 1 with combined nuclear *PAX1* and *DKK1* genes and mitochondrial D-loop variants: a case report

**DOI:** 10.3325/cmj.2025.66.56

**Published:** 2025-02

**Authors:** Siti Nornazihah Mohd Rosdi, Suzuanhafizan Omar, Mazira Mohamad Ghazali, Ab Rahman Izaini Ghani, Abdul Aziz Mohamed Yusoff

**Affiliations:** Department of Neurosciences, School of Medical Sciences, Universiti Sains Malaysia, Health Campus, Kubang Kerian, Kelantan, Malaysia

## Abstract

Chiari malformation type 1 (CM 1) is a rare and complex neurological condition. This congenital condition affects the lower posterior fossa, where the brain connects to the spinal cord. Although the exact cause of CM 1 remains unclear, genetic predisposition plays a considerable role in structural defects of the cerebellum. Here, we report on a 15-year-old female patient with CM 1 who exhibited both nuclear and mitochondrial genetic variants, a combination that has not been previously described. We identified a silent mutation in exon 2 (c. 556 G>A, p. Lys185 = ) of *PAX1* and a *DKK1* variant in intron 3 (548-3 *t* > C) in the nuclear DNA. We also screened the D-loop region of mitochondrial DNA as it exhibits a higher susceptibility to mutations than other mitochondrial DNA regions. Several hotspot variants were revealed, including those in positions 303-309 and 16519 (*t* > C), as well as some variants that had not been documented in MITOMAP. Our findings highlight the potential role of genetic alterations in D-loop in CM 1.

Chiari malformation type 1 (CM 1) is a neurological condition characterized by the herniation of the cerebellar tonsils more than 5 mm through the foramen magnum (1). It is often associated with syringomyelia and is more prevalent in women (2). CM 1 can be asymptomatic or present with neurological symptoms, including headaches (98% of patients), ocular disturbances, neck pain, vertigo, numbness, and scoliosis. Surgical treatment is required if severe neurological symptoms are present.

The cause of CM 1 remains unclear, but familial clustering and hereditary influence support the role of genetic factors (3). However, no single gene mutation has been identified as the primary cause of cerebellar defects. Environmental factors may also contribute.

Mitochondria house their own genetic material, mitochondrial DNA (mtDNA), which begins to replicate at the displacement-loop (D-loop), a non-coding region where two DNA strands are separated and stabilized by a third displaced strand (4). This D-shaped structure, evident in electron micrographs, also plays a crucial role in repairing DNA double-strand breaks through homologous recombination (4). The D-loop is a significant hotspot for genetic alterations in human cancers, which may impair mtDNA replication or transcription, resulting in mitochondrial dysfunction and increased cellular production of reactive oxygen species (ROS) (5).

The role of mtDNA in CM 1 remains incompletely understood and has not been previously explored in genetic pathways related to the condition. Given the rarity and complexity of CM 1, we focused on the D-loop region of mtDNA due to its higher mutation susceptibility. This additional testing aimed to provide a detailed genetic analysis, potentially shedding new insights on the genetic causes of CM 1 and guiding future research. Therefore, we report on a CM 1 patient with combined nuclear DNA (*PAX1* and *DKK1* genes) and mtDNA variants, which has not been previously documented in literature or reference laboratory databases.

## CASE REPORT

**Figure 1:**
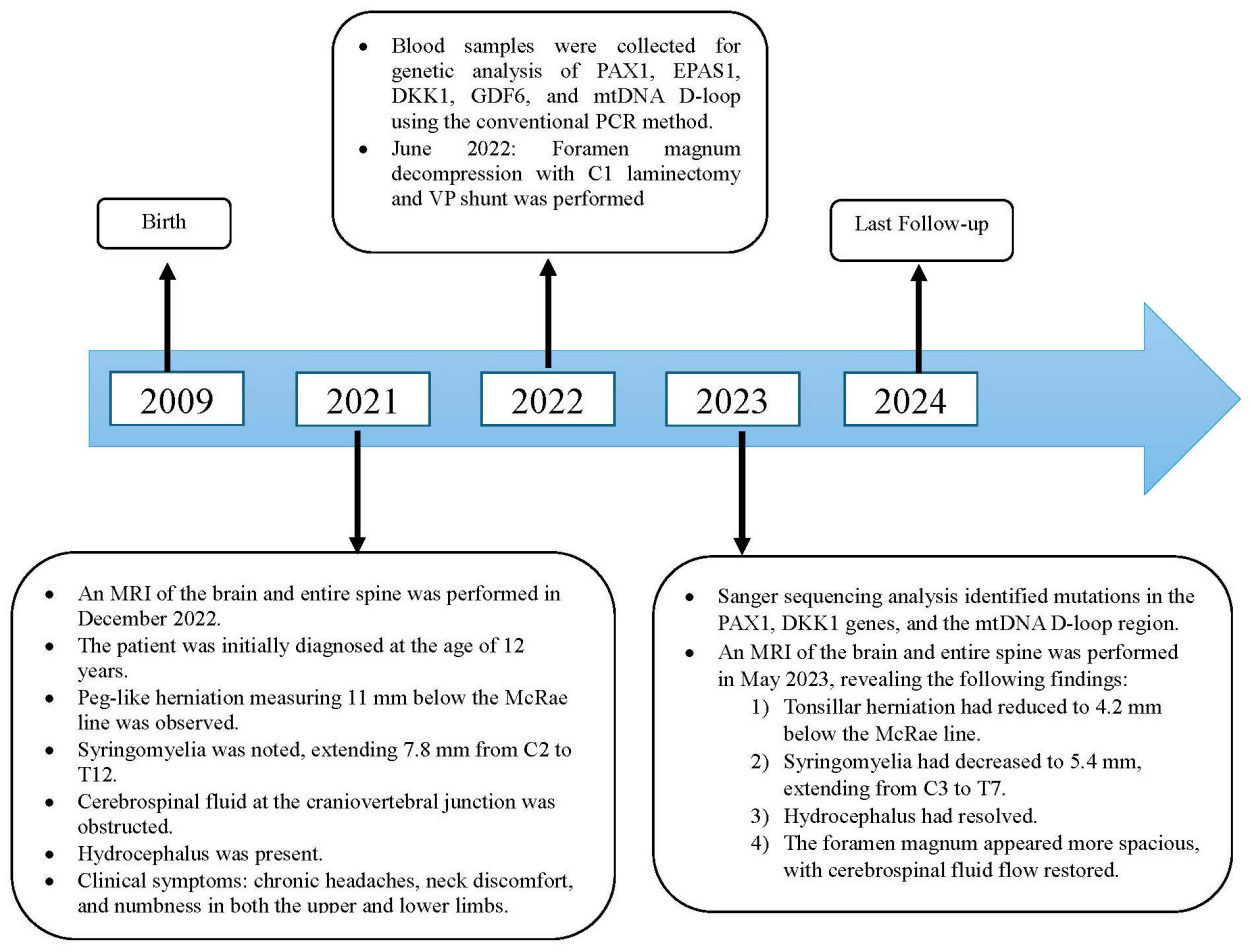
The timeline of events and interventions. mtDNA – mitochondrial DNA; PCR – polymerase chain reaction; VP – ventriculoperitoneal; MRI – magnetic resonance imaging.

**Figure 2:**
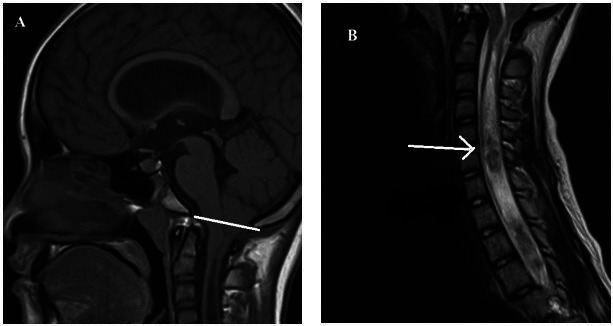
(**A**) Sagittal magnetic resonance imaging (MRI) showing Chiari malformation 1 (CM 1). The white line represents the McRae line connecting the basion and opisthion. (**B**) T2-weighted sagittal MRI displaying a cervical-thoracic syrinx, indicated by the white arrow.

A 15-year-old female patient (Figure 1), first diagnosed in 2021 at the age of 12, was evaluated at the Neuroscience Specialist Clinic of Universiti Sains Malaysia Specialist Hospital due to a history of chronic headache, neck discomfort, and bilateral upper and lower limb numbness. Magnetic resonance imaging (MRI) of the brain and entire spine revealed a peg-like herniation of the cerebellar tonsils through the foramen magnum, measuring 11 mm. The herniation led to crowding of the foramen magnum, with associated hydrocephalus and syringomyelia. The MRI findings confirmed the diagnosis of CM 1 (Figure 2). The MRI of the cervical spine revealed a long segment of hypointensity on T1/flair at the center of the spinal cord, which extended from the C2 level downward. It was consistent with cerebrospinal fluid (CSF) signal intensity and indicated a syrinx. Subsequently, the patient underwent a right ventriculoperitoneal shunt and foramen magnum decompression with C1 laminectomy. Postoperative MRI revealed a regression of tonsillar herniation to 4.2 mm below the McRae line, along with a reduction in syringomyelia to 5.4 mm. Additionally, hydrocephalus had resolved. The patient remains under regular follow-up.

### Detection of mtDNA D-loop variants

**Figure 3:**
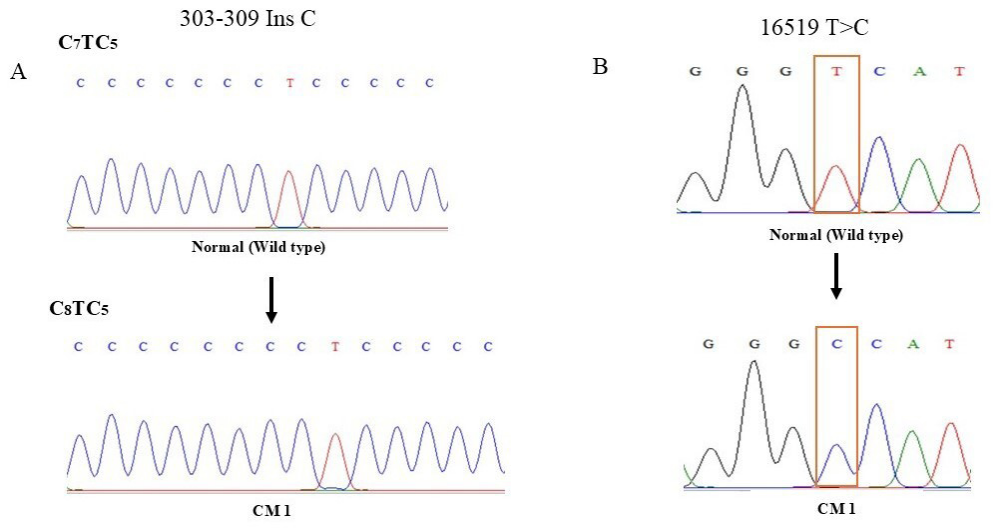
Mitochondrial DNA D-loop mutations in a patient with Chiari malformation 1. (**A**) The insertion of a cytosine at nucleotide position 303-309. (**B**) A homoplasmic mutation involving a T-to-C transition at nucleotide position 16519.

After providing informed consent, the patient underwent mtDNA D-loop mutation screening. Peripheral blood was collected for genomic DNA extraction, followed by the amplification of the mtDNA D-loop region using polymerase chain reaction (PCR) with human mitochondrial primers (5). Sanger sequencing was performed with the Big Dye Terminator cycle sequencing kit (Applied Biosystems, Foster City, CA, USA), and the data were analyzed with an Applied Biosystems 3730 Series Genetic Analyzer. Mutations and polymorphisms were confirmed through a repeated analysis of both strands. The results were compared with the Cambridge Reference Sequence of human mtDNA (NC_012920) from the MITOMAP database (http://www.mitomap.org). The screening revealed multiple mtDNA D-loop variants, such as those at positions 303-309 and 16519 (*t* > C), along with some variants not reported in MITOMAP (Table 1). Typical electropherograms are shown in Figure 3.

**Table 1:** Mitochondiral DNA D-loop variants detected in Chiari malformation 1 patients*

Somatic mutation	Homoplasmy/ heteroplasmy	Region	Novel/ reported	References
210 A>G	homoplasmy	HVS2	not reported in MITOMAP	present study
303-309 ins C	homoplasmy	CSB2	prostate cancer	(6)
489 *t* > C	homoplasmy	HVS3	prostate cancer	(6)
16031 C>G	homoplasmy	HVS1	not reported in MITOMAP	present study
16145 G>A	homoplasmy	HVS1	not reported in MITOMAP	present study
16181 A>G	homoplasmy	HVS1	not reported in MITOMAP	present study
16192 C>T	homoplasmy	HVS1	colonic mucosa	(7)
16291 C>T	homoplasmy	HVS1	diabetes type 2	(8)
16304 *t* > C	homoplasmy	HVS1	prostate cancer	(6)
16519 *t* > C	homoplasmy	ATT	prostate cancer	(6)

*Abbreviations: HVS2 – hypervariable segment 2; CSB2 – conserved sequence block 2; HVS3 – hypervariable segment 3; HVS1 – hypervariable segment 1; ATT – membrane attachment site.

### Detection of PAX1 and DKK1 gene variants

**Figure 4:**
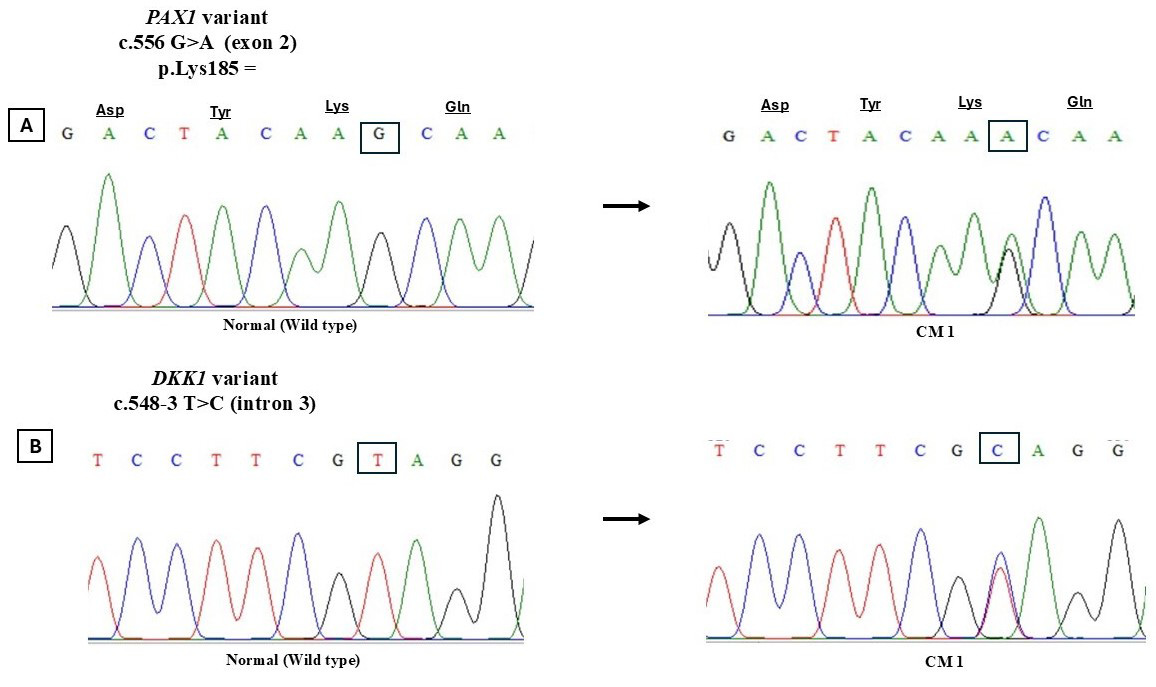
(**A**) Heterozygous *PAX1* variant in exon 2, c.556 G>A (p. Lys185 = ). (**B**) *DKK1* variant in intron 3, 548-3 *t* > C.

Genomic DNA was extracted with the same method for variant analysis in *PAX1* and *DKK1* genes, with PCR primers and conditions obtained from McGaughran et al (9) and Marello et al (3), respectively. Sanger sequencing identified a synonymous transition mutation (c.556 G>A) in the *PAX1* gene at codon 185 (AAG) in exon 2, maintaining the amino acid lysine (Figure 4). A heterozygous variant (c.548-3 *t* > C) was detected in the intron of the *DKK1* gene.

## DISCUSSION

CM 1 is largely sporadic, though familial aggregation and hereditary influence suggest a genetic predisposition. A contributing factor may be the underdevelopment of occipital somites from the paraxial mesoderm, involving the *PAX* gene family (10,11). *PAX* genes encode transcription factors critical for pattern formation during vertebrate embryogenesis. Specifically, *PAX1*, located on chromosome 20p11.2, is essential for vertebral segmentation and development (9). A disrupted *PAX1* function has been associated with vertebral fusion and re-segmentation issues (9). In this study, we identified a silent mutation in exon 2 of *PAX1* (c. 556 G>A), which did not alter protein structure. However, silent mutations may still impact protein levels and function by influencing mRNA stability, splicing, or translation efficiency (12). Macaya et al reported a *de novo* silent mutation (c.3612 TCA>TCC [p. Ser1204Ser]) in Treacher Collins syndrome, an autosomal dominant craniofacial disorder linked to the *TCOF1* gene. This mutation caused splicing defects and abnormal mRNA processing (13).

*DKK1*, a 29 kDa protein in the Dickkopf (*DKK*) family, inhibits the Wnt signaling pathway, which is critical for vertebrate embryogenesis, including head formation, skeletal development, and limb patterning (14). It encodes a secreted ligand that binds to LRP6 receptors, blocking Wnt signaling and pathway activation (15). Disruptions in this pathway can lead to craniofacial and neural malformations that contribute to CM 1. An altered *DKK1* expression affected craniofacial and central nervous system (CNS) development in animal studies. For example, *DKK1* overexpression in *Xenopus* embryos caused enlarged anterior head structures, while anti-DKK1 antibody injections led to microcephaly (16). Although the *DKK1* intronic variant (Intron 3 c.548-43 *t* > C) identified in this study lacked functional evidence, its potential impact on other cell types or developmental stages cannot be ruled out. Intronic mutations can become pathogenic if they activate cryptic splice sites, leading to exon skipping or intronic sequence inclusion in mature transcripts (9). An example includes β-thalassemia, where an intronic mutation disrupts pre-mRNA splicing, impairing β-globin protein production (17).

Currently, no studies have examined mitochondrial genome mutations associated with CM 1. Research on CM 1 has primarily focused on nuclear DNA due to its established role in genetic predispositions influencing CM 1 development. This study also concentrated on mtDNA, often referred to as the second genome, targeting the D-loop region for its high mutation susceptibility (5). In addition to *PAX1* and *DKK1* variants, the patient exhibited mtDNA D-loop mutations at positions 309 and 161519. The D-loop, a non-coding regulatory region spanning 1124 bp (position 16024-576), controls mtDNA replication and transcription, containing hypervariable regions HVI (16024-16383), HVII (57-372), and HVIII (438-574) (5). This region is a hotspot for mutations linked to various disorders, including cancers and neurodegenerative diseases. Notably, the C7TC5 mononucleotide repeat sequence (303-316/318) frequently exhibits deletion or insertion mutations, termed mitochondrial microsatellite instability. mtDNA mutates over ten times faster than nuclear DNA due to its proximity to the electron transport chain and the absence of protective histone proteins, which increases its vulnerability to ROS (5). These mutations impair ATP synthesis and elevate ROS production, inducing oxidative stress and mitochondrial dysfunction. Understanding mtDNA's involvement in CM 1 may allow insights into its pathogenic mechanisms.

In the context of CM 1, mitochondrial dysfunction can disrupt the development or stability of brain structures, including the cerebellum. Mutations in the mtDNA D-loop may increase cellular ROS production, contributing to oxidative stress in neural cells. This could, in turn, affect mtDNA replication and transcription.

In conclusion, we present the first reported case of a patient with CM 1 exhibiting a dual genetic variant (nDNA and mtDNA), which has not been previously documented in literature or reference laboratory databases. This case highlights a potential for further investigation into the relationship between mtDNA and CM 1, suggesting a new direction for further research. CM 1 is closely linked to CNS anomalies, as it involves the structural displacement of cerebellar tonsils into the spinal canal. Furthermore, mitochondrial dysfunction associated with alterations in the mtDNA D-loop region may play a role in CNS anomalies, potentially disrupting energy metabolism and increasing oxidative stress levels.

This case report enhances our understanding of CM 1 by exploring the involved genetic factors, particularly nuclear and mitochondrial variants, and highlighting the role of mtDNA mutations in the development of the condition. These findings could guide future research into genetic predisposition and potential therapeutic strategies.
